# Angular Light, Polarization and Stokes Parameters Information in a Hybrid Image Sensor with Division of Focal Plane

**DOI:** 10.3390/s20123391

**Published:** 2020-06-16

**Authors:** Francelino Freitas Carvalho, Carlos Augusto de Moraes Cruz, Greicy Costa Marques, Kayque Martins Cruz Damasceno

**Affiliations:** 1 Department of Electronics and Computation, Universidade Federal do Amazonas, Manaus 69077-000, Brazil; carlosamcruz@ufam.edu.br (C.A.d.M.C.); greicy@ufam.edu.br (G.C.M.); kayquedamasceno18@gmail.com (K.M.C.D.); 2 INFRAERO Empresa Brasileira de Infraestrutura Aeroportuária, Manaus 69041-000, Brazil; 3 SIDIA Instituto de Ciências e Tecnologia, Manaus 69055-035, Brazil

**Keywords:** light polarization, micro-polarizer filter array, CMOS image sensor (CIS), Stokes parameters, division-of-focal-plane, SPICE

## Abstract

Targeting 3D image reconstruction and depth sensing, a desirable feature for complementary metal oxide semiconductor (CMOS) image sensors is the ability to detect local light incident angle and the light polarization. In the last years, advances in the CMOS technologies have enabled dedicated circuits to determine these parameters in an image sensor. However, due to the great number of pixels required in a cluster to enable such functionality, implementing such features in regular CMOS imagers is still not viable. The current state-of-the-art solutions require eight pixels in a cluster to detect local light intensity, incident angle and polarization. The technique to detect local incident angle is widely exploited in the literature, and the authors have shown in previous works that it is possible to perform the job with a cluster of only four pixels. In this work, the authors explore three novelties: a mean to determine three of four Stokes parameters, the new paradigm in polarization cluster-pixel design, and the extended ability to detect both the local light angle and intensity. The features of the proposed pixel cluster are demonstrated through simulation program with integrated circuit emphasis (SPICE) of the regular Quadrature Pixel Cluster and Polarization Pixel Cluster models, the results of which are compliant with experimental results presented in the literature.

## 1. Introduction

Recently, there have been increases in the demand for new multimedia resources of three-dimensional content, particularly in images, games, movies and augmented reality. In this context, capturing 3D information or depth sensing is essential for many applications including object and material classification [1,2,3], navigation [4,5], image polarization contrast in biological tissues [6,7,8,9], improved vision in haze conditions [10] and diagnosis in oncology [11,12], different views of the same image [13,14], facial recognition in the surveillance area [15,16,17,18], atmospheric remote sensing and other applications. Therefore, the search for improvements in depth sensing and 3D image capture has become an important target in the field of image sensors.

It has been shown that through the aid of capturing the local angle of incidence and the intensity of light received, the whole 3D information of the recorded scene can be reconstructed. In order to provide solutions to this problem, numerous methods for 3D picture image capture have been proposed within the literature, including Time-of-Flight (ToF) [19,20], multi-apertures [21,22,23], Talbot’s diffraction pixels set [24,25,26], division of amplitude [27], division of focal plane microgrid (DoFP) with polarization pixel filters [24,28,29,30] and quadrature pixels [9,25,26]. Disadvantages of the previous techniques include additional laser source and time to process the laser signal in ToF, a large amount of micro-lens that require a very large imager array in the case of multi-apertures image sensors, and a large number of pixels to decode angle variations that involves non-trivial post-processing in the case of Talbot’s pixels. In order to avoid such issues, recent approaches propose the use of two sets of different pixel clusters [31,32,33,34], the polarization pixel cluster (PPC) and the quadrature pixel cluster (QPC). All these techniques have limitations with their benefits and disadvantages, however, in this work, only the most relevant characteristics of the sets of polarization and quadrature will be taken into account.

**Figure 1:**
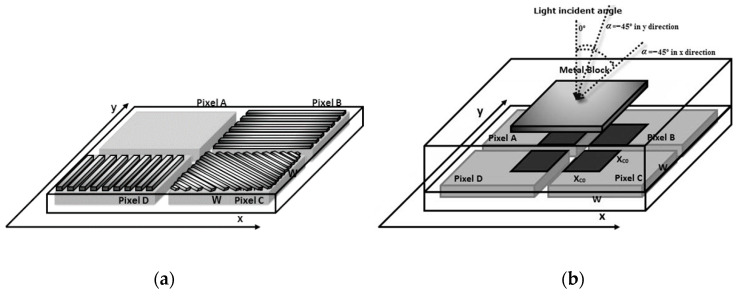
(**a**) Polarization Pixel Cluster (PPC) [31]; (**b**) Quadrature Pixel Cluster (QPC) [31].

According to the proposition of [31,32,34], detecting and measuring the angle of incidence and polarization of light employs two sets of different pixel clusters, the PPC and the QPC. The PPC is composed of four pixels: A, B, C and D, as shown in Figure 1a. It is built with an unshielded pixel used to detect local light intensity that serves as reference value; a pixel with horizontally organized microwire which might be strongly sensitive to 90° polarized light, a pixel with vertical grids strongly sensitive to 0° polarized, and a 45° grid pixel to determine the Stokes parameters. The PPC provides high sensitivity to both light polarization and local incident angle [31,32,34]. It is shown to be much more sensitive to the variation of the angle of incidence than the classic Talbot pixel. To determine the incident angle of the local light, only the horizontal and vertical PPC grated pixels are required. However, positive and negative angles produce similar PPC output, and consequently, an extra technique is required to determine the sign of the light incidence angle, whether positive or negative.

In order to determine the angle of incidence sign, the QPC is employed. It produces a reasonably weak response, however, could be very linear and proportional to the variation of the light incident angle. The QPC, Figure 1b, is much less touchy to incidence angle variation than PPC, but its output is not symmetric around zero, as is PPC. Therefore, this cluster can be employed to determine the light incident angle sign.

As shown above, in terms of imager resolution and consumption of additional resources, the solution proposed in [31] is so far the best known solution for local intensity and angle detection. However, the configuration of the PPC and QPC pixel components can be further optimized in order to achieve improvements in terms of imager resolution. It shows the possibility to build a more optimized pixel cluster with the same functionalities presented in [31] using only four pixel components out of the PPC and QPC set of pixels.

In this paper, the behavior of the photocurrents in the QPC and PPC component pixels as a function of pixel grating, local light intensity, polarization and incident angle is evaluated in a simulation program with integrated circuit emphasis (SPICE), and the results are employed as the basis of the new compact pixel cluster that is able to replace the QPC and PPC. It is worth noting that tools and seminal analyses of the approach herein presented were already reported by the authors in [35,36,37] where details of an optimized pixel cluster were presented and discussed, however, such a configuration was unable to provide the data needed to determine Stokes parameters, as will be discussed. The new configuration of a hybrid quadrature-polarization pixel cluster (HPC) presented in this work is able to perform all the functions performed by previous solutions, and to provide the necessary data to determine the first three Stokes parameters. Moreover, the minimum resolution of an imager array employing the proposed pixel cluster is 75% higher than that of previous solutions, and it may be extended to its complete potential resolution, as will be shown along this text.

The paper is organized as follows: the background concepts of pixel details of the photocurrent and topology model used, pixels sensitive to polarization and incidence of light, and a short principle about Stokes parameters and degree of linear polarization (DoLP) are presented in Section 2. The proposed hybrid macropixel is described at the end of Section 2. The simulation outcome and the discussions showing the viability of the proposed solution are exhibited in Section 3. The conclusions drawn from this work are given in Section 4.

## 2. Materials and Methods

This section describes the basic concepts used all through this work.

### 2.1. Operating Principles of Micropolarizer Array-Based Polarization Sensors

**Figure 2:**
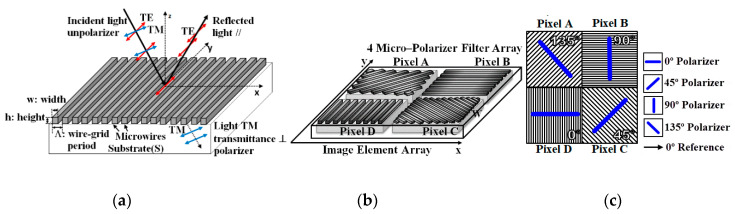
(**a**) Three-dimensional view of a division of focal plane polarization sensors on the imaging plane. (**b**) A micropolarizer array-based polarimeter with orientation. (**c**) Top view of the microgrid with the four main filters.

The first simulated cluster was the PPC. It works based on the principle of polarization by absorption, also known as division of focal plane (DoFP). When the light strikes with its electric field vector parallel to the thin wire, electric currents are generated along the wire and the energy of light is absorbed, simply because the waves are absorbed by the microwires [33,38,39]. If the electric field is perpendicular to the grids, the maximum level light is transmitted, as shown in Figure 2a.

The sensor array of photo detectors is covered with an array of wire-grid polarizer matched microwire polarization filters. A pattern of the polarization filter array is shown in Figure 2b,c. It consists of four distinct filters which can be offset way of by 45 degrees: 0°, 45°, 90° and 135° (the same angle of −45°).

### 2.2. Photodiode Model

**Figure 3:**
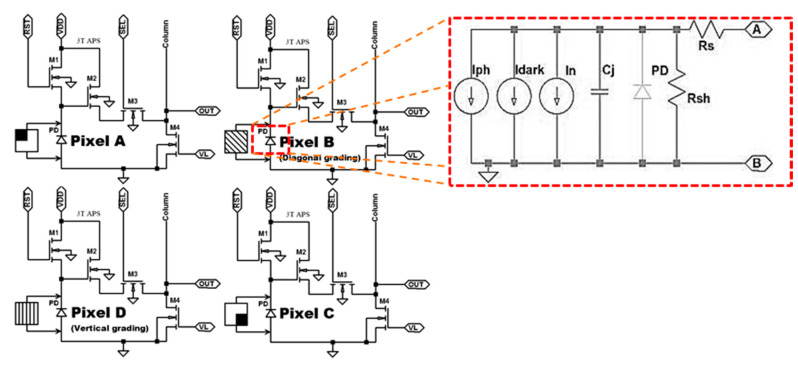
Schematic of a single hybrid quadrature-polarization pixel cluster (HPC) pixel in the 3T-APS topology designed in a 0.18 µm standard TSMC CMOS process with the electrical model of a photodiode in red dashed detail.

All the pixels of the clusters have the same schematic topology, presented in Figure 3 known as of the 3T-APS. BSIM3v3 simulations have been executed with an active pixel sensor in a pattern of 6-metal 1-poly, complementary metal oxide semiconductor (CMOS) 0.18 μm TSMC technology. The pixels of these clusters operate in the linear mode that provides excellent sensitivity in the direction of low light intensities, and a reduced dynamic range towards high light intensities. In the SPICE circuit electronic simulator, the pixel photodiode (PD) of the 3T-APS was modeled consistent with the schematic detailed on the right of Figure 3, in red dashed detail, wherein *R_s_* is the PD series resistance, *R_sh_* is the PD shunt resistance, *I_ph_* is the PD photocurrent, *I_dark_* is the PD current level at dark situation, *C_j_* is the PD junction capacitance. For the analyses herein presented, the noise contributions, current *I_n_* in the Figure 3, was not taken into account. The photocurrent *I_ph_* is the current generated by the photons reaching the depletion region of the PN junction [40], which in this case is a function of the pixel grating and of the light polarization, incident angle and intensity.

The light output information is transduced and treated as a voltage or current signal. It was chosen to work with voltage as the output signal, wherein all stages of the linear mode CMOS active pixel sensor (APS) operation cycle may be observed. The linear mode CMOS APS operating cycle can be divided into three special intervals: the reset time, the exposure time and the sampling time detailed in [40]. The principal parameters are given in Table A1 in the Appendix A.

### 2.3. Polarization Pixel Cluster

The light irradiated along the transmission axis of a polarizer with division of focal plane reaches the surface of the photodetector PD according to the extended Malus’s law [33], in which the maximum irradiance is given by Equation (1):

(1) $$I_{ph}\left(\psi ,\alpha \right)=I\left(0\right)\cos^{2}\left(\psi \right)kA\cos\left(\alpha \right)+I_{dark}$$

where *ψ* is the angle between the maximum electric field and the transmitter axis of the analyzer, and *I*(0) is the initial irradiation incident at the grid. In the case of the wire-grid polarizer, the transmission axis of the grid is perpendicular to the microwaves, as shown in Figure 2b. To achieve this polarization target using the photocurrent model shown in Figure 3, the value of *I_ph_* is modeled to be proportional to the square of the cosine of the polarization angle *ψ*. Moreover, a cosine factor multiplier *k* proportional to the area of the photodiode *A* and angle of incidence *α* was added according to Lambert’s law [31], as an additional dark current *I_dark_*.

The pixel A of the PPC cluster is completely unshielded and thus exposed to the entire external incident light, as shown in Figure 1a. Apart from the grating scheme, the circuit topology of each PPC pixel is the same as that presented in Figure 3. In Pixel B with horizontal grids, the maximum transmission will take place when the light is polarized at 90° and in Pixel D with vertical grids, the maximum transmission will occur when the light is at 0°, as can be seen in Figure 1a. In the grid arranged at −45°, the maximum transmission will occur when the polarization of the light is 90° displaced, that is, at 45° with respect to the chosen orientation of the grids, which is exactly the Pixel C in Figure 1a.

### 2.4. Quadrature Pixel Cluster

The second simulated cluster was the QPC illustrated in Figure 1b. This is a 3D view of the cluster, consisting of a block of metal on the top of the four photodiodes in such a way that the area which receives the incidence of light is proportional to the light incident angle.

As detailed in [25], on this cluster, the direction of the incident light is determined by the unshaded area, i.e., by the area of the photodiode that has incidence of light. Knowing that the total area is given by *A* and the areas without shade are given by *A*_ushD_, that is, the areas that receive light will be given by Equation (2), where *α_x_* and *α_y_* are angle on *x*-axes and *y*-axes, respectively. The other parameters are given in Table A1, in the Appendix A.

These mathematical equations were embedded in the *I_ph_* photocurrent model proportional to the area *A*, thus enabling the simulation of the QPC according to a topology just like that shown in Figure 1b.

(2) $$A_{\mathrm{ushD}}=A-\left\{X_{D0}+\left[\left(T_{im\varepsilon }+T_{M}\right)\cdot \tan\left(\arcsin\left(\frac{n_{\mathrm{ar}}}{n_{\varepsilon }}\sin\alpha_{x}\right)\right)\right]\right\}\;\times \left\{X_{D0}+\left[\left(T_{im\varepsilon }+T_{M}\right)\cdot \tan\left(\arcsin\left(\frac{n_{\mathrm{ar}}}{n_{\varepsilon }}\sin\alpha_{y}\right)\right)\right]\right\}$$

### 2.5. Stokes Parameters and Degree of Polarization

The parameters *S*_0_, *S*_1_, *S*_2_ and *S*_3_ are referred to as the Stokes polarization parameters for a plane wave [1,5,33]. The polarization state of an electromagnetic wave can be defined more easily through this set of parameters. They were first introduced in optics by Sir George Gabriel Stokes in 1852. The Stokes parameters are real quantities and are simply the variable observables of the polarization ellipse, and consequently, the optical field [41,42].

The first Stokes parameter *S*_0_ is the overall light intensity with the unfiltered intensity value [43]. The parameter *S*_1_ describes the amount of linear or vertical linear polarization. The parameter *S*_2_ describes the quantity of linear polarization +45° or −45° (135°). Finally, the parameter *S*_3_ describes the amount of right or left circular polarization contained in the beam. It is possible to observe that the four Stokes parameters are expressed in terms of intensity, and again it is emphasized that the Stokes parameters are measurable real quantities and provide the polarization properties of light [41,42].

The Stokes parameters are computed based on the intensity measurements from the photodiodes and are presented by Equations (3)–(7) in which *I*_0°_ is the intensity of the light after passing through a linear polarizer of 0°, and *I*_90°_, *I*_45°_ and *I*_135°_ are the intensity after light passing of 90°, 45° and 135°, respectively. Thus, the parameters can be given by:

(3) $$S_{0}=Intensity=I_{tot}$$

(4) $$S_{0}=I_{0{}^{\circ}}+I_{90{}^{\circ}}$$

(5) $$S_{1}=I_{0{}^{\circ}}-I_{90{}^{\circ}}$$

(6) $$S_{2}=I_{45{}^{\circ}}-I_{135{}^{\circ}}$$

(7) $$S_{3}=I_{RHC}-I_{LHC}$$

The circular polarization element *S*_3_ describes the excess of left-hand circularly polarized portion over the right-hand circularly polarized portion, which will not be used in this work.

In the works of [28,37,43,44], they show that the Stokes parameters can be expressed by the following equations:

(8) $$S_{1}=2I_{0{}^{\circ}}-I_{tot}$$

(9) $$S_{2}=2I_{45{}^{\circ}}-I_{tot}$$

With the Stokes parameters, it is possible to determine the degree of polarization (DoP) of any optical signal directly through its *S* components. The DoP quantifies the fraction of the optical signal that is really polarized, and the DoP of a totally polarized optical signal is equal to the unit, while the DoP of a non-polarized optical signal is zero, given by Equation (10).

If a beam of light is linearly polarized, the circular and elliptical polarizations are zero. Its degree of polarization is therefore regularly known as the linear polarization degree (DoLP). The degree of linear polarization of a light beam is described by Equation (11).

In order to obtain all optical properties of partially polarized light, three parameters are important to know: wave intensity, degree of linear polarization (DoLP) and polarization angle (AoP). The vector AoP can be represented by way of Equation (12).

(10) $$DoP=\sqrt{\left(S_{1}^{2}+S_{2}^{2}+S_{3}^{2}\right)}/S_{0}$$

(11) $$DoLP=\sqrt{\left(S_{1}^{2}+S_{2}^{2}\right)}/S_{0}$$

(12) $$AoP=\left(1/2\right)\cdot \arctan\left(S_{2}/S_{1}\right)$$

**Figure 4:**
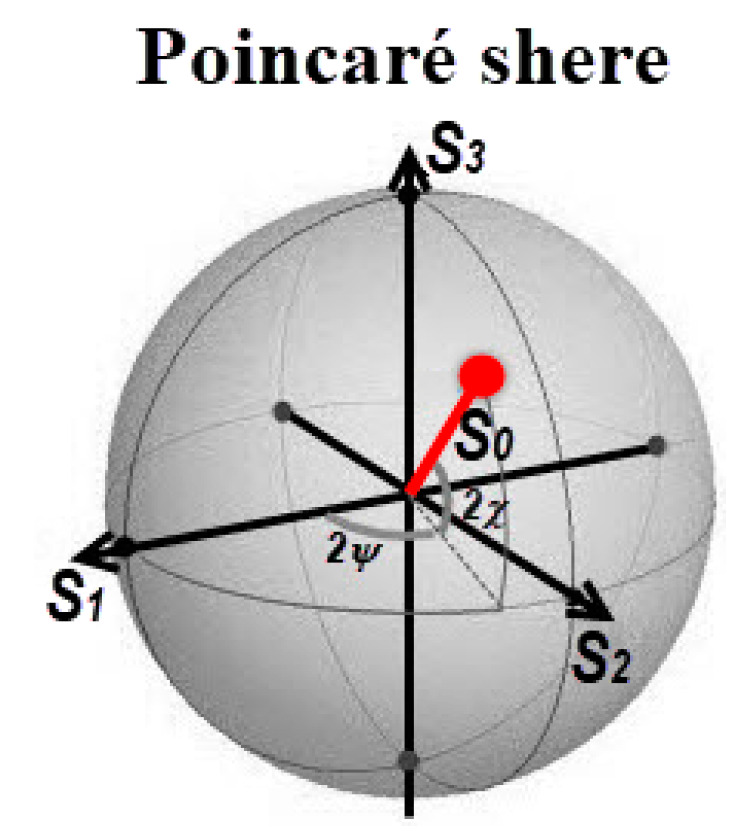
Poincaré sphere representation.

An alternative way to visualize the Stokes parameters is through the Poincaré sphere shown in Figure 4, used to view the four independent *S* components as points on or within the sphere [5,41,45].

In Figure 4, it is possible to observe the polarization azimuthal angle *ψ* and the ellipticity angle *χ*. The surface of the Poincaré sphere is typically used to give a top-level view representation of all the possible polarization states of completely polarized light. In this situation, the polarization azimuthal angle *ψ* and the ellipticity angle *χ* can be expressed in terms of the Stokes parameters following Equations (13) and (14):

(13) $$\sin2\chi =S_{3}/\sqrt{S_{1}^{2}+S_{2}^{2}+S_{3}^{2}}$$

(14) $$\tan2\psi =\left(S_{2}/S_{1}\right)$$

### 2.6. The Hybrid Pixel Cluster Proposed

**Figure 5:**
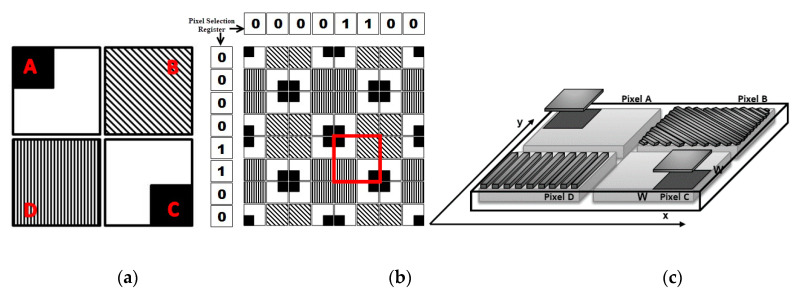
(**a**) Layout of proposed HPC-hybrid polarization-quadrature pixel cluster sensor. (**b**) Example of an array with 16 HPC units with one selected in the red square. (**c**) A 3D view of the HPC.

Employing different pixel clusters as in [31] reduces the resolution of the imager sensor, thus more compact pixel clusters are desirable. To purposefully mitigate this issue, a new approach using features of both the PPC and QPC is proposed in this paper. The new HPC pixel cluster is a hybridization of the two preceding pixel clusters. The proposition is to replace the 90° sensitive pixel B in Figure 1a by a 45° sensitive pixel and update both the light intensity detection pixel A of the PPC cluster and the pixel C by two pixels of QPC in opposite location, therefore resulting in the cluster of pixels shown in Figure 5a.

The two HPC grated pixels still have the same functions as those of the PPC cluster, one is a filter out to 0° of polarization and the other to 45°. These two PPC pixels were chosen in order to enable the calculation of the third Stokes parameter *S*_2_ as described in Equation (6) or (9). With the previous solution of the work of [37] in which the 0° and 90° pixel were used, it was not possible to determine *S*_2_. Whereas the two other pixels A and C have two different functions, the first is to compose two different QPC clusters and the other is to detect the local light intensity. An instance of an array pattern shaped by 16 devices of the proposed HPC cluster is presented in Figure 5b. All the four pixels of the proposed pixel cluster have the schematic topology, shown in Figure 3. A 3D view of the HPC is presented in Figure 5c.

Besides being a more compact pixel cluster, the other main advantage of the HPC is its capability to detect the light intensity using the pixels A and C. For the same light intensity level, the output results of pixels A and C will be dependent upon the local light incident angle. Nevertheless, either the sum or averages of the two output results are independent of the local light incident angle in the complete angle detection range. The outcomes presented in [25] show that the complete angle detection range for the QPC cluster is between −45° and 45°.

The shielded area within the pixels A and C reduces their sensitivity, and for a given light intensity, the average of the two outputs will be lower than the output of the unshielded pixel A of the PPC in Figure 1a. Although there is a reduction in sensitivity, it is not so bad, as it will be shown, and does not restrain the usability of the proposed procedure for local light intensity detection.

The intensity pixel A within the PPC, in Figure 1a, was employed in [5] and [43] in order to normalize the response of the polarization pixels B, C and D. The pixel cluster presented in [43] has the same structures of the HPC proposed in this work, but it does not have the ability to determine the incident angle sign as does the QPC. However, it is possible to determine the local light intensity in a polarization pixel even without the specialized light intensity detection pixel as inside the PPC.

The proposed HPC produces a virtual light intensity response in the middle of the pixel cluster in the position shown by the red square in Figure 5b instead of producing it in one of its pixels as in the case of the PPC. Despite the reduced sensitivity of light intensity, the resolution of a matrix employing the HPC cluster is 75% higher than that of one employed the solution proposed in [34]. This happens because the information of a single point in an imager employing the cluster of [34] requires seven pixels to be completely represented, resulting in 1/7th of the total array resolution, whereas using the proposed cluster solution requires only four pixels, resulting in 1/4th of the total array resolution.

The problem with the reduced light intensity sensitivity of proposed technique will be discussed in the next section.

## 3. Results and Discussion

### 3.1. Circuit Simulation Data

**Figure 6:**
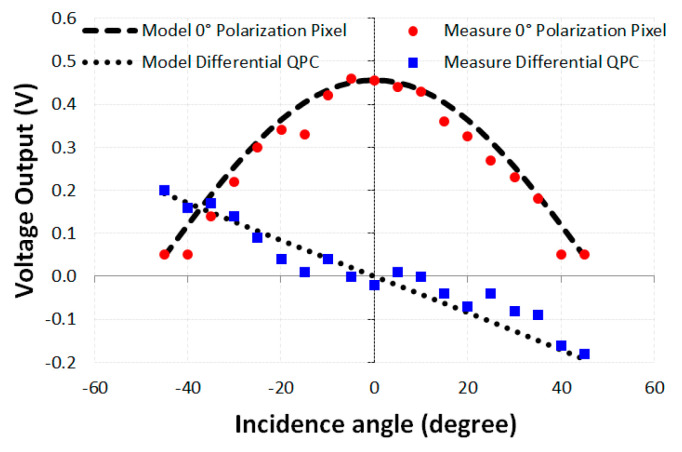
Pixel voltage versus incidence angle variation of differential (*V*_out_A_ − *V*_out_C_) quadrature pixel of QPC, dotted plot, and 0° polarization pixel D of PPC, dashed plot, illustrating the angle detection technique in simulation compared to experimental data from [31].

The SPICE simulation results of pixel D with vertical grids, in Figure 5a, are presented in Figure 6. In this situation, the maximum transmission takes place when the light is polarized at 0° and the higher output level takes place at 0° of incidence angle. The simulation results of the proposed model are compliant with those of experimental reports found in the literature, as can be verified by comparing the dashed and dotted plots against the experimental results extracted from [31]. Such comparison is possible because two of the four pixels that compose the HPC are similar to two of the pixels of the QPC cluster, whereas the other two are similar to two of the pixels of the PPC cluster. Therefore, similar pixels even in different clusters might yield the same output results. Thus, the new proposed HPC model can be regarded as quite reliable, as will be discussed in the following statistical analysis.

The PPC together with the QPC results for the light polarization of 0° and range of incident angle varying from −45° to 45° are plotted in Figure 6 with a step of 5°, where the dotted plot is the result of the differential output of the QPC pixels A and C (*V*_out_A_ − *V*_out_C_) and the dashed plot is the output result of the PPC pixel D with 0° polarization. It is worth noting that using the difference between the output of QPC pixels A and C results in higher signal swing than using each one individually [36]. The difference between the two output signal yields and output range from −0.2 to 0.2 V allows the measurement of the incident angle from −45° to 45°. This information complements the PPC symmetry deficiency. These results are consistent with those demonstrated in [31]. Similar results are generated in the proposed HPC.

The Minitab statistical software, Version 16 was used to manage the data. The R^2^ ratio between the 0° PPC pixel data simulated with our model and the experimental results in the literature was 0.969. The value of Pearson’s correlation coefficient between these data was 0.984. Knowing that the coefficient value is 1 means that the correlation between the variables is perfectly positive. If this value has a value of −1, it means that the relationship between the variables is perfectly negative. If this value is 0, it means that the variables are independent of each other. As the value obtained was very close to the unit with a relative error of 1.6%, we can conclude that the simulated data presented good accuracy in relation to the experimental data, therefore, the model becomes reliable. For the QPC data, the value of R^2^ was 0.934 among the simulation data in our proposed model in relation to the experimental data obtained from [31]. The value of Pearson’s correlation coefficient was 0.966. As the value obtained was also very close to the unit with a relative percentage error of 3.4%, this result also makes the model reliable due to this good accuracy.

When doing a linear regression between the PPC data, to obtain a relationship of the type *y* = a*x* + b, considering *y* as the simulated data and *x* the measured data from the literature, we obtained *y* = 0.940*x* + 0.042, emphasizing that in the ideal case this equation would be *y* = *x*. This result can be considered satisfactory to validate the PPC model. Applying the linear regression to the QPC data, considering the same convention of the previous paragraph for *y* and *x*, we obtained *y* = 1.08*x* − 0.0103. Again, this result is considered satisfactory for the validation of the QPC model.

The results of the 45° polarization sensitive of the proposed HPC model are the same as the PPC; that is, when the light polarization was 45°, maximum light transmission was obtained, and when it was −45°, parallel to the grids, the minimum light transmission was obtained. The result of the pixel sensitive to the 0° polarization of the HPC was similar to that of the PPC, shown in Figure 6, since they have the same configuration of sensitivity to the polarization of 0°. With regard to the proposed HPC solution, the output voltage values for the difference between pixel A and pixel C show that these pixels behave in the same way as QPC pixels. The difference between the two pixel output indicates the angle of light incidence.

**Figure 7:**
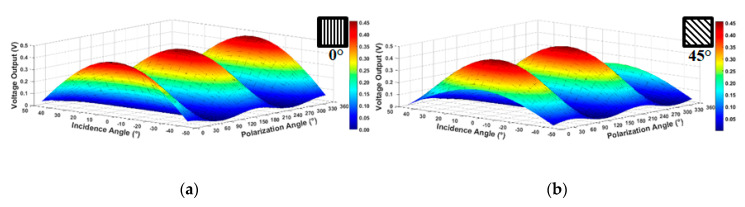
Pixel output voltage of intensity response of the 3T APS (**a**) pixel D of 0° polarized and (**b**) pixel B of 45° polarized versus sign of incidence angle and polarization angle.

Now, Figure 7 depicts a three-dimensional simulated pixel level voltage output for each pixel as a function of the sign of incidence angle and polarization angle to the pixels D (0° polarizer) and B (45° polarizer) of the HPC, respectively. In these plots, the incident angle varies from −45° to 45° with 5° step and polarization angle varies from 0° to 360° with a step of 5°.

It is possible to see that in a particular situation in which the polarization angle is 0° in Figure 7a with variation only of the incidence angle, it produces precisely the concave plot shown in Figure 6.

**Figure 8:**
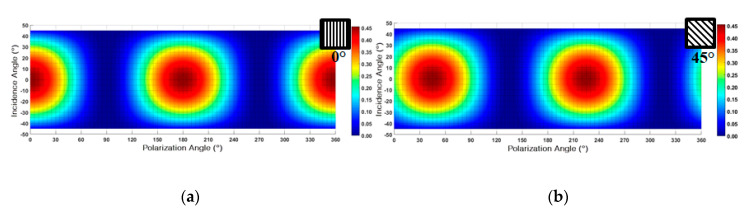
Two-dimensional distribution map of the output voltage of the light intensity pixel and a corresponding voltage line profile (right column) of (**a**) 0° polarization and (**b**) 45° polarization as a function of the incidence angle and the polarization angle.

The simulated voltage distribution map on the focal plane to light polarized at 0° is shown in Figure 8a and of light polarized at 45° in Figure 8b. This is a two-dimensional distribution graph of the output voltage of the light intensity pixel and a corresponding voltage line profile of 0° polarization (left) and 45° polarization (right) as a function of the incidence angle and the polarization angle. In this figure it is possible to examine the symmetry around the axis of light incidence at 0°, and consequently, the QPC is essential to determine the incidence sign.

**Figure 9:**
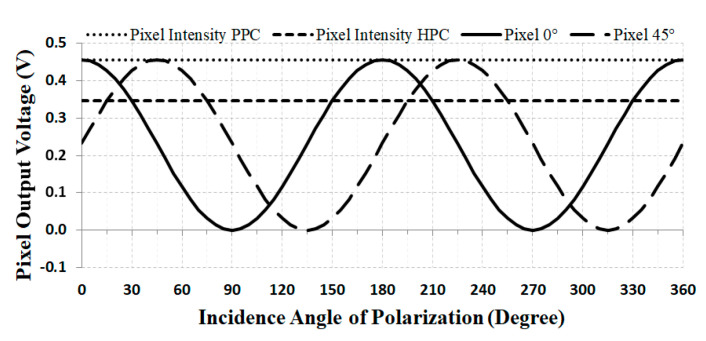
Pixel output voltage of intensity response of: PPC Pixel A, HPC Pixel A with normal angle of incidence to the sensor, linearly polarized to 0° and 45°.

In a specific situation where the polarization angle varies from 0° to 360° with step of 5° and only the normal incident angle is taken into account, i.e., the perpendicular beam in the surface of the sensor, the results match those displayed in Figure 9. These results are also similar to the experimental reports of [46] considering the higher light transmission. The light intensity plots are shown in Figure 9, where it is possible to compare the difference between the outputs of pixel A of PPC of 0.456 V and that of pixel A of the proposed HPC of 0.344 V. The HPC voltage level is lower than that of the PPC because of the metal shielding that reduces the pixel light incident area and therefore its sensitivity. This issue can be overcome, for example, by the sum of two or four adjacents pixels.

Using the graphic Poincaré sphere to visualize different types of polarized light and using Equations (3), (8) and (9) in which only the intensity pixels *I*_tot_, *I*_0__°_ and *I*_45__°_ are used, it is possible to determine the Stokes parameters *S*_0_, *S*_1_ and *S*_2_. Although the HPC does not produce the same intensity levels of PPC, it is possible to produce a proportional *I*_tot_ value through normalization.

Our proposed cluster model in which the 90° grated pixel was replaced by the 45° grated pixel is analyzed. Thus, all parameters are acquired using Equations (3), (8) and (9). However, in this model, there is no combination of *I*_0__°_ and *I*_90__°_ to obtain *I*_tot_, and, consequently, the Equation (4) cannot be used, as it was the work of [46] which used these two pixels to determine the value of *I*_tot_. Although the intensity value is lower than in the unshielded pixel (PPC pixel A), the following equations can be employed to correct this distortion: *I*_tot_ = *cte* × *I′*_tot_, in which *I′*_tot_ is the light intensity in the partially shielded pixels of the HPC cluster and *cte* is a constant that represents the ratio between the light sensitive area of a fully exposed pixel and a partially shielded pixel. For example, if the metal shield covers 25% of the pixel light sensitive area, *cte* is determined by *I*_tot_/*I′*_tot_ = 1/0.75 = 1.333.

The plots in Figure 9 show the voltage output signal of the 0° and 45° grated pixels, the intensity pixel the PPC and of the intensity of the HPC, considering normal light incidence with light polarization angle varying from 0° to 360° with steps of 5°. In this case where *V*_tot_ = 0.456 V and *V′*_tot_ = 0.344 V are equivalent to 75.4% of the total incident light with a percentage error of 0.53%, resulting in a *cte* value of 1.326. The real value of this parameter can only be experimentally determined, as it depends on the sensitivity conditions of the photodiode, the size and mismatches of the manufacturing process of the metal shielding, as well as the losses in the dielectric medium of SiO_2_.

### 3.2. 3D Simulation Results

**Figure 10:**
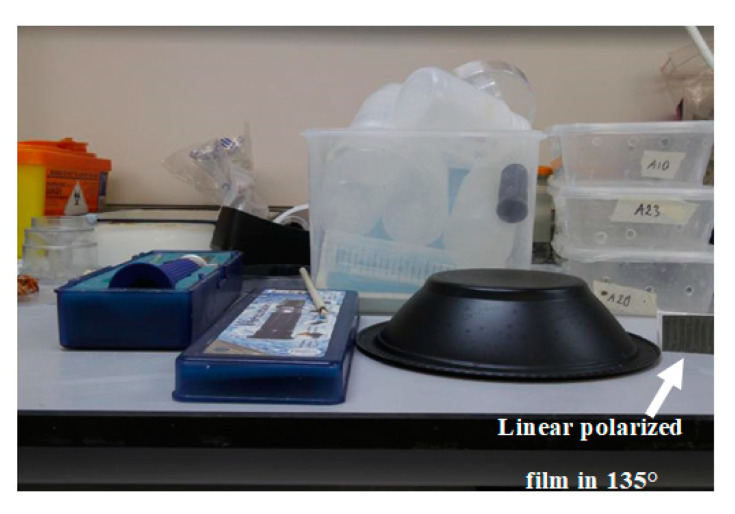
Image with different plastic materials including a black plastic bowl. On the right side, a linearly polarized film plastic at 135° was also added for testing.

To show the feasibility of the proposed HPC concept, polarized images at 0° and 45°, as well as an image equivalent to 3/4 of the total intensity equivalent to the partially shielded, were acquired. With these three images, the equivalent operation of the proposed pixel cluster is presented and discussed. Figure 10 shows an image with different plastic materials including a black plastic bowl. On the right side of the black bowl, a linearly polarized film plastic at 135° was included for testing.

**Figure 11:**
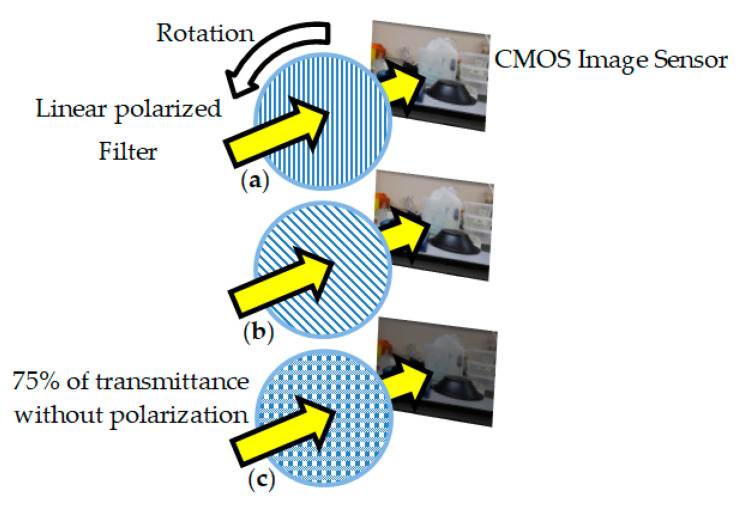
(**a**) Polarized image detection system with a polarizer on the front of the image sensor to 0°. (**b**) The polarization filter was rotated with 45° step to get 45° polarized image. (**c**) Representation of an image with 75% of transmittance without polarization.

For the acquisition of the polarized images, a 2304 × 3456 pixels CMOS polarimetric photographic sensor was used, with the addition of a linear polarization filter in the front position of the image acquisition device, as can be seen in an illustrative way in Figure 11. A set of two images was collected using a Canon EFS lens with 15–85 mm with a linear polarization filter (72CP, Tiffen) oriented at 0° and 45° in front of the CMOS sensor device and properly rotated at the appropriate angles to obtain the two polarizations desired, as seen in Figure 11a,b. All lighting, positioning, framing and sensor settings were kept constant for each set of images to ensure that there were no differences between the images. A third image was obtained without polarization and a computational treatment was performed with MATLAB using digital image processing to obtain an image with 75% light intensity transmittance. Figure 11c is an illustrative manner to represent that the light was attenuated.

**Figure 12:**
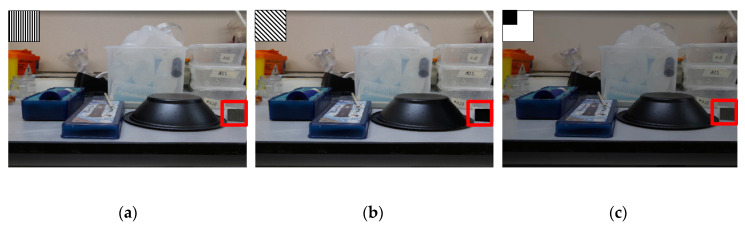
(**a**) Polarized images of the scenario with polarizations 0°, (**b**) 45° and (**c**) 75% of intensity without polarization.

The images of the scenario with polarizations 0° and 45° can be observed in Figure 12a,b, which are equivalent to a whole image by HPC type D and B pixels, shown previously in Figure 5a, i.e., both *I*_0°_ and *I*_45°_ images, respectively. In Figure 12c, it has the image equivalent to pixels A and C in Figure 5a, in which there is a metal covering that blocks 25% of the photodiode, herein named *I′*_tot_. These images were used as inputs, simulating what we would obtain with the HPC shown in Figure 5a, and will be used to calculate Stokes parameters.

There are small noticeable differences among the images when visual inspection is carried out, but changes are observed in the black bowl that shows slight visual variations. It is emphasized here that, although human beings have a well-developed vision that detects variations of color and brightness in several orders of magnitude, they are unable to detect polarization information [32], and therefore, we do not observe many differences between the images. The most noticeable visual change is in the 135° polarized plastic film on the right side of the bowl, which has a darker color in Figure 12b which is the 45° polarized image. The plastic film is polarized at 135° and the image is polarized at 45°, that is, the image is polarized with an absolute difference of 90° with respect to the film. Thus, the polarizations are perpendicular to each other, and consequently, the film features a color approaching black. In the image of Figure 12a, the plastic film is polarized at 135° (−45°), but the image is polarized at 0°, i.e., the image is polarized with an absolute difference of 45° with respect to the plastic film, which implies an intermediate color between white and black. In Figure 12c, there is an image with 3/4 of the pixel intensity level of the original image without polarization filter, which corresponds to 75% of the total incident light intensity.

It is noticeable that the variations in intensity along the figure of the black plastic bowl are minimal in most of the image and the shape of the figure is difficult to determine simply with the image of two-dimensional intensity as shown in Figure 10.

**Figure 13:**
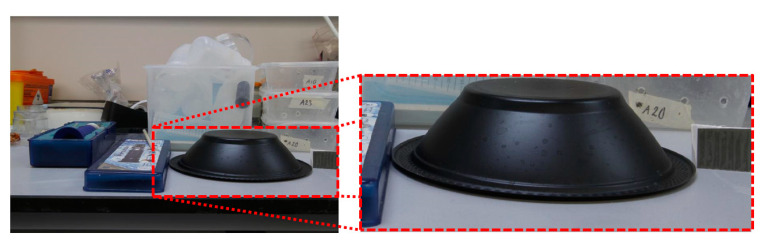
Image highlighting the black bowl, a piece of the plastic cover on the left and the 135° polarized film with dimensions 700 × 1938, which are used for the formation of the three-dimensional image.

A 700 × 1938 resolution image was chosen that included the bowl, a piece of plastic cover on the left and the 135° polarized film on the right, as highlighted in Figure 13. This image was chosen with a tangent cut to the upper part of the bowl, as shown in Figure 13, to improve the formation of the three-dimensional image, as will be shown next.

**Figure 14:**
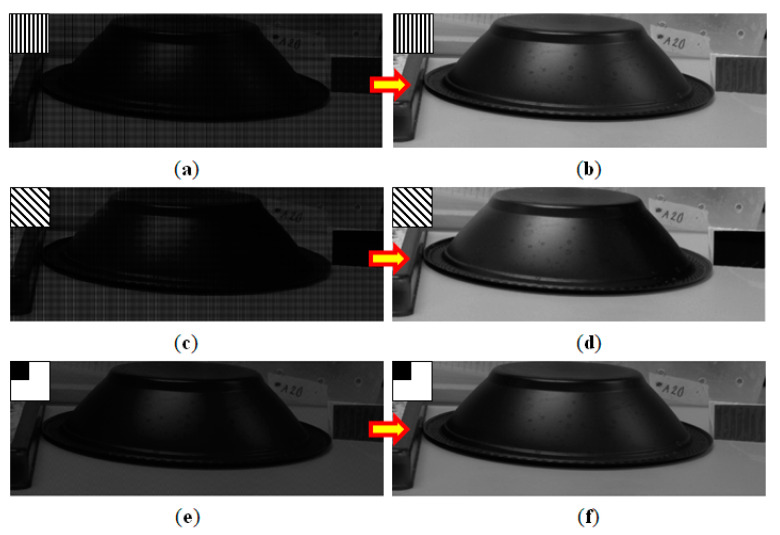
(**a**) Image of 0° polarized pixels with zeros in other positions. (**b**) The same results of 0° polarized pixels without the zero points. (**c**) Image of 45° polarized pixels with zeros in other positions. (**d**) Image of 45° polarized pixels without zero points. (**e**) Image of partially covered pixels (75% free) with zeros in other positions. (**f**) Image of the partially covered pixels without zero points.

The MATLAB R2015a software was used to perform the processing of polarized images, running on a DELL computer model INSPIRON N4110 with Windows 10 operating system (64 bits) and Intel Core i3-processor 2350M CPU @ 2.3 MHz with 8 GB of RAM. Initially, the image of Figure 12a was cut and transformed into a computational grayscale. To obtain the points equivalent to those of 0° polarization (pixel D of the HPC in Figure 5a), the pixel value of intensity was collected in those specific positions and zero values were assigned to the other positions, resulting in Figure 14a. Considering the image without zeros, we have the result shown in Figure 14b, which is a 0° pixel polarized image without the zeros points.

Then, the image in Figure 12b was also cut and transformed into a grayscale. To obtain the points equivalent to those of 45° polarization (pixel B of the HPC in Figure 5a) in the same way as before, the pixel value of intensity was collected in those specific positions and zero values were assigned to the other positions, resulting in Figure 14c. With the removal of the zero points, the image of Figure 14d was obtained.

To obtain the third image, a similar process was performed in which the image of Figure 12c was cut and transformed into grayscale to obtain pixels A and C of the proposed HPC pixel of Figure 5a. Similar to the previous procedure, the other pixels are set to zero, resulting only in pixels of 75% intensity, as can be seen in Figure 14e. Again, the zero points were removed, with Figure 14f as the result.

Thus, in the images of Figure 14 on the right side: Figure 14b,d,f, the images *I*_0°_, *I*_45°_ and *I′*_tot_ of the black bowl are shown, respectively.

**Figure 15:**
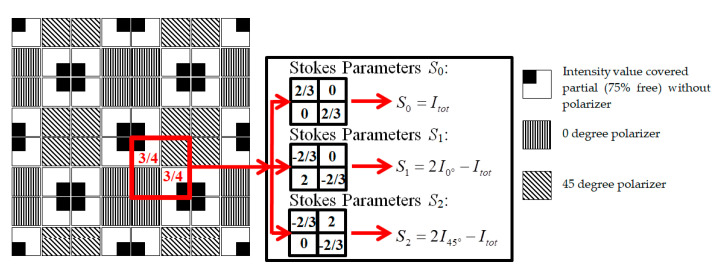
Block diagram of a complete focal plane polarization imaging system.

In the image sensor shown in Figure 15, a 2 by 2 pixel HPC cluster is addressed and accessed simultaneously. In the 2 by 2 calculation window, one pixel records the projected polarized image at 0 degree (*I*_0°_), another records the polarized image projected at 45 degrees (*I*_45°_) and two partially covered pixels record the intensity image unfiltered (*I′*_tot_). Polarimetric parameters are estimated by reading all four pixels in parallel and scaling them individually at the periphery, that is, far from the image matrix.

With the weighted multiplications shown in Figure 15, the Stokes parameters *S*_0_, *S*_1_ and *S*_2_ are obtained, which is equivalent to using Equations (3), (8) and (9), previously given in Section 2.5.

**Figure 16:**
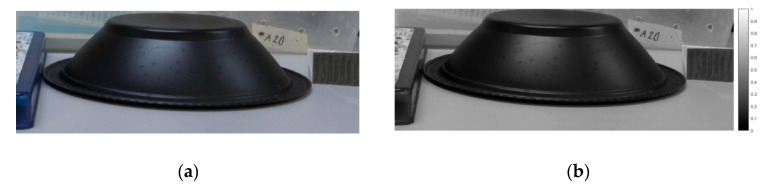

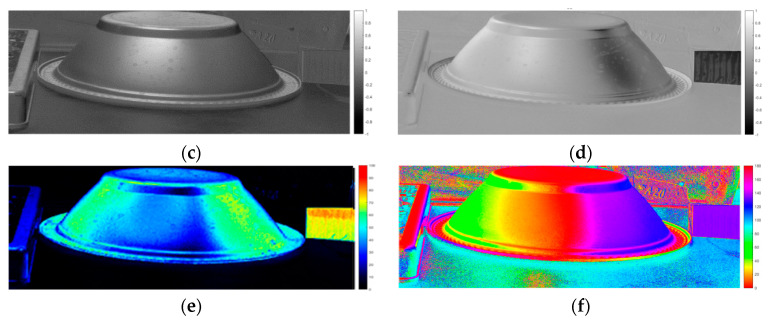
(**a**) Original image. (**b**) Image of the Stoke Parameter *S*_0_. (**c**) Image resulting of Stoke Parameter *S*_1_. (**d**) Stoke Parameter *S*_2_. (**e**) Degree of Linear Polarization (DoLP) with pseudo-color. (**f**) Angle of Polarization (AoP) with pseudo-color.

The original image of the scene cut and already in grayscale can be seen in Figure 16a next to the result of the intensity information in Figure 16b obtained by the procedure explained above, in which the values close to zero represent the darkest areas and values close to the unit represent the brightest areas, as can be seen in the gray scale bar on the right side of the image. Thus, it has the intensity equivalent *I*_tot_, which is equal to the first Stokes parameter *S*_0_ from Equation (3).

In Figure 16c, the second Stokes parameter *S*_1_ was obtained, in which the brighter portions correspond to those of horizontal polarization, that is, they are the areas that present more polarization oriented at 0°, whose values are close to the value +1 and the darker values represent the areas with vertical polarization (90°) with the values closer to −1, as can be seen in the grayscale bar on the right side of the image.

It is possible to observe in Figure 16d, the third Stokes parameter *S*_2_ where the brighter portions correspond to the 45° polarization areas, that is, they are the areas that present more polarization oriented at 45° whose values are close to the +1 value and the darker values represent the areas with polarization at 135°, with values closer to −1, as can be seen in the grayscale on the right side of the image. It is observed that the 135° polarized filter that was added on the right side of the image presented the values closest to −1 in contrast to the other pixels of the image, that is, the area with the closest 135° polarization which corresponds to the same −45° polarization. It is also observed that the plastic bowl presents the largest variation range, from −1 to +1, which means the polarization ranges from −45° to +45°.

DoLP normalized parameter for the scenario was obtained using Equation (11), as shown in Figure 16e. In order to increase the visibility of certain resources in the scene, a false color, also called pseudocolor [41,47,48], was applied to these images, resulting in Figure 16e, similar to what was done in the works of [49,50] where the bias percentage was shown as a different color. Pixels with black color represent a low degree of linear polarization and with those colors closer to red represent a high degree of linear polarization for two images, according to the color scale on the right side of Figure 16e. The linear polarization filter located on the right side of Figure 16e also has a high DoLP value due to the intrinsic properties of the filters.

In Figure 16f, there is the representation of the linear polarization angle (AoP) with the application of the pseudocolor for the scenario, where 0° of linear polarization is presented in red, 90° of linear polarization is presented in light blue and again 180° linear polarization is shown in red. It is observed that the plastic bowl has a wide range of polarization angles, but these linear polarizing angles along the plastic figure exhibit smooth variations due to the curvature of the figure since the light reflected from a surface changes its polarization state based on the angle of reflection. Thus, observing the state of polarization allows the recovery of the normal surface [47]. The polarization-sensitive sensor captures image information on the intrinsic environment, i.e., surface curvature and refractive index represented in the data space of the polarization angles.

It is observed that the variations in intensity along the figure of the black plastic bowl are minimal in most of the image and the shape of the figure is difficult to determine simply with the image of two-dimensional intensity as shown in Figure 13.

In Figure 16e, in which the false color was applied to improve the visual perception of the image, the difference between the plastic bowl and the polarized linear filter is more evident when compared to the rest of the image, that is, it presented high percentages degree of polarization.

As the region of the image of the plastic bowl showed high variations in polarization angles (AoP) and presented greater degrees of linear polarization (DoLP), this image was chosen with a section tangent to the top of the bowl to improve the formation of the 3D image, as will be shown next.

The information obtained from the polarization image sensor was used for the reconstruction of a 3D image using a single camera, instead of what is usually done, in which several cameras are used to obtain various points of view of the same image and, consequently, form the three-dimensional image. The degree of polarization captured by the CMOS polarization image sensor is directly related to the angle of incidence of the incoming light and the normal surface of the image object [46]. The polarization measurements of the CMOS Image Sensor () are converted to normal surface information of the object contained in the image. The DoLP shown in Figure 16e provides an estimation of the surface profile of the plastic bowl, as well as the polarized plastic film and some of the plastic cover on the left.

**Figure 17:**
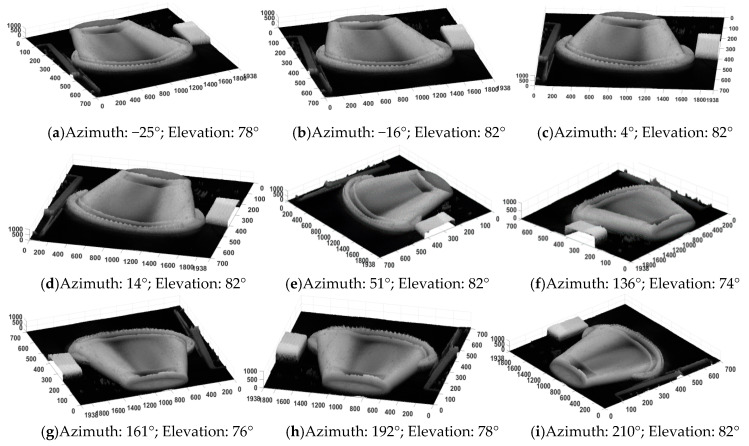
Three-dimensional surface reconstitution of a bowl using the degree of linear polarization (DoLP) in several points of view (**a**–**i**).

In Figure 17, there is a three-dimensional reconstruction of this scenario, in which the normalized amplitude of the DoLP was multiplied by a thousand to improve the relationship between the dimensions of the width, thickness and height of the image. The three-dimensional image reconstruction from the DoLP in the CIS is presented from different viewpoints. It is possible to see the formation of the 3D image of the bowl, the polarized film and the piece of plastic cover, which have high relief in relation to the base of the image (in black). This result is important, as it is similar to those obtained in [46,47], which obtained reconstructions of an image of a plastic horse and a PET bottle, respectively, using the same principles of this work. In this case, it was possible to reach the state-of-the-art in the area of polarized sensors, extraction of Stokes parameters from an image and reconstitution of a three-dimensional image from these.

## 4. Conclusions

In this paper, a compact hybrid pixel cluster was presented with the capacity to detect local light intensity, local incident angle, and local light polarization, which also enables it to determine Stokes parameters. The proposed hybrid pixel cluster embeds the functionality of the two-pixel clusters previously presented in the literature, the polarization pixel cluster and the quadrature pixel cluster. The greatest advantage of the proposed solution is its potential to perform the same functions of the previous pixel cluster solutions with 75% improved resolution. The biggest downside of the proposed solution is the need for employing more than one pixel to determine the local light intensity level, however, this was not a problem to determine the Stokes parameters. Analyses over a set of images captured with a polarizer system on the front of the image sensor are presented and discussed, showing the expected 3D image reconstruction capabilities of an imager employing the proposed pixel cluster. The simulation results show that the proposed solution, based on simple modification of well-established pixel cluster topologies, results in an improved CMOS image sensor for depth sensing and 3D image reconstruction purposes at no additional fabrication cost of the integrated circuit.

## Appendix A

**Table A1:** Important sensor parameters.

Parameter	Value
Technology	0.18 µm standard TSMC CMOS process
Pixel size (*A*)	5 µm × 5 µm
Pixel type	Photodiode 3T APS
Width/Length of the CMOS Transistors: *W*_1_/*L*_1_, *W*_2_/*L*_2_, *W*_3_/*L*_3_, *W*_4_/*L*_4_	0.4/0.2, 5/0.2, 5/0.2, 0.4/0.2 µm
Fill Factor	40%
Capacitance of the photodiode junction (*Cj*)	20 fF
Dark Current	0.1 fA
Supply Voltage	1.8 V
Height of the metal M (*Tim**ε*)	6.360 µm
Thickness of the metal M (T*m*)	1.720 µm
Refractive index of air (*n_air_*)	1
Refractive index of SiO_2_ (*n**_ε_*)	1.46
Widths of the shaded regions of diodes under normal illumination (*X*_A0_*, X*_B0_*, X*_C0_*, X*_D0_)	2.3 µm
Light intensity	500 klux = 732 W/m^2^ [*λ* = 555 nm]
